# Androgen Receptor and Histone Lysine Demethylases in Ovine Placenta

**DOI:** 10.1371/journal.pone.0117472

**Published:** 2015-02-12

**Authors:** Ellane R. Cleys, Jennifer L. Halleran, Vanessa A. Enriquez, Juliano C. da Silveira, Rachel C. West, Quinton A. Winger, Russell V. Anthony, Jason E. Bruemmer, Colin M. Clay, Gerrit J. Bouma

**Affiliations:** 1 Department of Biomedical Sciences, Animal Reproduction and Biotechnology Laboratory, Colorado State University, Fort Collins, Colorado, United States of America; 2 Department of Animal Sciences, Colorado State University, Fort Collins, Colorado, United States of America; II Università di Napoli, ITALY

## Abstract

Sex steroid hormones regulate developmental programming in many tissues, including programming gene expression during prenatal development. While estradiol is known to regulate placentation, little is known about the role of testosterone and androgen signaling in placental development despite the fact that testosterone rises in maternal circulation during pregnancy and in placenta-induced pregnancy disorders. We investigated the role of testosterone in placental gene expression, and focused on androgen receptor (AR). Prenatal androgenization decreased global DNA methylation in gestational day 90 placentomes, and increased placental expression of AR as well as genes involved in epigenetic regulation, angiogenesis, and growth. As AR complexes with histone lysine demethylases (KDMs) to regulate AR target genes in human cancers, we also investigated if the same mechanism is present in the ovine placenta. AR co-immunoprecipitated with KDM1A and KDM4D in sheep placentomes, and AR-KDM1A complexes were recruited to a half-site for androgen response element (ARE) in the promoter region of *VEGFA*. Androgenized ewes also had increased cotyledonary VEGFA. Finally, in human first trimester placental samples KDM1A and KDM4D immunolocalized to the syncytiotrophoblast, with nuclear KDM1A and KDM4D immunostaining also present in the villous stroma. In conclusion, placental androgen signaling, possibly through AR-KDM complex recruitment to AREs, regulates placental VEGFA expression. AR and KDMs are also present in first trimester human placenta. Androgens appear to be an important regulator of trophoblast differentiation and placental development, and aberrant androgen signaling may contribute to the development of placental disorders.

## Introduction

Proper placentation is required for normal fetal development and nutrient transport to the fetus. Placentation begins as trophoblast cells from the implanting embryo differentiate to form cytotrophoblasts and syncytiotrophoblast [1]. Trophoblast differentiation and invasion during the first trimester is essential for establishing placental vascularization for prenatal support throughout gestation [1–3]. While the placenta is widely recognized as an endocrine organ, sex steroids act on placental cells to regulate placental development and function. For example, estrogen signaling has been shown to regulate trophoblast differentiation and invasion in a hypoxic environment of first trimester primate placenta [4–7]. Estrogen receptor alpha (ESR1) localizes primarily in human cytotrophoblast and differentiating cytotrophoblast cells [8–9], and estradiol produced by the placenta regulates trophoblast invasion by increasing matrix metalloproteinase (MMP) activity and promoting angiogenesis in baboon [7,10]. Although estrogen contributes to placentation via trophoblast differentiation and uterine spiral artery remodeling [4,7], surprisingly little is known about the role of placental androgens considering maternal androgen levels rise during normal pregnancy, and abnormal androgen levels observed in women with polycystic ovarian syndrome and preeclampsia are associated with compromised pregnancies [11–14].

During human pregnancy, maternal peripheral plasma concentrations of androstenedione and testosterone increase approximately two-fold and four-fold, respectively [11,12]. A similar increase in serum testosterone is seen in pregnant cows [15]. Plasma levels of androgens return to non-pregnant levels a few days postpartum, suggesting the placenta as a source of androgen production [11]. Importantly, androgen receptor (AR) is present in trophoblast cells in human, cow, goat, and pig placentas [16–19] and has been characterized recently in differentiating bovine invasive trophoblast giant cells [16]. In the human placenta, AR is localized to syncytiotrophoblast and vascular endothelial cells, although it also has been reported in cytotrophoblasts and invasive extra villous trophoblasts [17,19,20]. Interestingly, linkage analysis and AR polymorphisms are associated with preterm birth and spontaneous abortions, respectively [21,22].

Ligand bound AR functions as a dimer by binding androgen response elements (AREs: GGA/TACAnnnTGTTCT) in promoter regions of target genes for transcription initiation [23]. Androgen signaling in non-placental tissues has been shown to regulate cell proliferation, invasion, and angiogenesis [24–26]. Recently, AR has been shown to complex with lysine demethylase 1 family and Jumonji-C domain containing histone demethylases (KDMs) to regulate AR target genes [27–29]. KDMs function to regulate gene expression by demethylating mono-, di-, and tri-methylated lysines on histone N-terminal tails [30], increasing DNA access to sex hormone receptors and coactivators for transcription initiation [31]. Additionally, KDMs bind to and are co-activators of *AR* and *ESR1* transcription [27,31–33], and increase expression of sex hormone target genes [27–29,34,35]. While KDMs appear to play a prominent role in regulating target gene expression, they have not been identified in placental tissue.

The overall goal of this study was to identify a role for androgen signaling in placental cells by focusing on the AR, and we hypothesize that AR binds to KDM’s in trophoblast cells. To this end, we used three approaches: (1) we examined changes in placentome morphology and gene expression following a well-established model of prenatal androgenization in ewes that were treated with testosterone propionate from gestational day GD30 to GD90 [36]. Previous studies clearly demonstrate this model for polycystic ovarian syndrome in women leads to abnormal fetal programming and intrauterine growth restriction [36], abnormal placental development [37], and offspring with reduced fertility, and hyperinsulinemia [38,39]. Furthermore, although there are many possible gene targets, we focused our attention on genes known to be targets of AR in cancer cells and play a role in placental processes such as proliferation, migration and angiogenesis. (2) We determined the localization of AR and KDM’s, as well as their interaction, in sheep placentomes. (3) Finally, because AR localization in placental tissue has been described, we assessed the localization of KDM’s in human first trimester placental tissue.

## Materials and Methods

### Prenatal Androgenization and Placentome Collection

All experiments were approved by the Colorado State University Institutional Animal Care and Use Committee. Fourteen crossbred ewes were bred and randomly assigned to one of two groups, control or testosterone propionate (TP) treated. Control ewes received intramuscular injections of 2 mL vehicle (cottonseed oil) biweekly from gestational day GD30 to GD90 (n = 7). TP treated ewes received biweekly intramuscular injections of 100 mg of testosterone propionate resuspended in 2 mL of cottonseed oil as previously described (n = 7) [36]. On GD90, ewes were anesthetized, uteri were surgically removed, fetal lambs (S1 Table) were weighed, and the placentas were collected. Individual placentomes from each placenta were isolated and classified as either type A, B, C, or D based on gross morphology [40,41]. For placentome types A, B, and C, the cotyledon was separated from the caruncle and processed individually; for type D placentomes this was not possible. The 5 placentomes closest to the umbilicus were used for analysis.

Of the five placentomes closest to the umbilicus, two were randomly selected for fixation in ice cold 4% paraformaldehyde (PFA) overnight at 4°C. The following day, PFA was removed and the placentomes were stored in 70% ethanol at 4°C until embedded in paraffin blocks. Remaining cotyledon and caruncle tissue were snap frozen in liquid nitrogen and stored at -80°C until processed. Prior to RNA, DNA, and protein isolation, samples were pulverized in liquid nitrogen. Pulverized tissue was stored at -80°C until placed directly in appropriate lysis buffer for each isolation protocol. To avoid any possible prejudices in data due to fetal sex, placentomes from both male and female fetuses from each treatment group were used for analysis. For all experiments, unless otherwise noted, there were 4 type A placentomes from 4 controls, 3 type A placentomes from 3 testosterone treated ewes.

### First Trimester Human Placenta Samples

Human first trimester placental samples were donated from three elective terminations from anonymous, non-smoking, non-drug using patients, with written consent in accordance with the Colorado State University Institutional Review Board policy. Samples were stored in sterile PBS upon collection and were transferred to ice cold 4% PFA upon receipt. Samples were stored overnight at 4°C in PFA, then transferred to 70% ethanol at 4°C until embedded in paraffin blocks. For immunohistolocalization, three 11.5 weeks of gestation samples were examined in triplicate. Tissue sections of 5μm thickness were taken from the center of paraffin blocks.

### DNA Isolation and ELISA of Global DNA Methylation

Genomic DNA was isolated using approximately 10 mg of pulverized ovine cotyledon and caruncle tissue using Wizard Genomic DNA Isolation Kit (Promega, #A1125) per manufacturer’s protocol. Concentration and purity of DNA was determined using a NanoDrop 1000 Spectrophotometer (NanoDrop Technologies, Wilmington, DE). DNA was stored at -80°C until used for analysis. MethylFlash Methylated DNA Quantification Kit (Epigentek, P1034) was used for detection of 5-methylcytosine as per the manufacturer’s protocol. 100 ng of DNA from each sample was used in duplicate. Standards were used in duplicate from 0.5 ng/μL to 10 ng/μL to quantify 5-methylcytosine in samples. Colormetric readings at 450 nm were recorded on a model 680 microplate reader (BioRad). Average absorbance values for the negative control were subtracted from each sample to obtain normalized values. A linear equation was calculated from standard curve dilutions. Normalized values were divided by the slope of the linear equation multiplied by two to obtain the amount of 5-mehtylcytosine present in each sample (ng of 5-mC). Statistical analysis was performed using ANOVA followed by Tukey pairwise comparison (Minitab 16).

### Protein Isolation and Western Blot

Approximately 2 mg of pulverized ovine placentome tissues were added directly to 2mL of ice cold lysis buffer containing 0.48M Tris pH7.5, 10mM EGTA pH8.6, 10mM EDTA pH8.0, and 0.1% (w/v) PMSF and protease inhibitor. Samples were sonicated on ice for 5 minutes and centrifuged at 10,000 rpm for 10 minutes at 4°C. Supernatant was collected and frozen prior to protein concentration analysis using a Bradford standard curve (BioRad). Supernatant was diluted in 6x SDS-DTT loading dye with 0.375M Tris pH6.8, 4M glycerol, 0.21M SDS, 0.6M DTT, and 0.06% (w/v) bromophenol blue. β-mercaptoethanol (1.75 μL) and water was added to reach a final concentration of 4.29 μg/μL of protein in 35 μL (150 μg total protein). Samples were boiled for 5 minutes after addition of β-mercaptoethanol, then electrophoresed at 95 volts in 10% Tris-HCL polyacrylamide gels (BioRad). For ESR1 blots, 10% Tris polyacrylamide gels were made using 10.2% acrylamide/bis solution (BioRad), 3.5mM SDS, 4.5mM TEMED (BioRad), and 43.3mM APS. Protein was electrophoresed in an ice cold running buffer containing 50mM Tris, 384mM glycine, and 7mM SDS. Protein was transferred onto 0.2 μm nitrocellulose membranes (Protran) for 1 hour at 200 milliamps at 4°C in transfer buffer containing 2mM Tris, 150 mM glycine, 5M methanol, and 3.5mM SDS. After protein transfer, blots were blocked for non-specific binding with 2% milk-TBST for 1 hour at room temperature. After blocking, blots were washed with TBST and left overnight at 4°C with primary antibody diluted in 2% milk-TBST. Antibodies used for Western blot analysis are listed in Table 1 with their respective dilutions. After incubation with primary antibody, blots were washed in TBST and incubated for 1 hour at room temperature with secondary antibodies, goat-anti-rabbit-HRP (Abcam ab6721, 1:1000) or goat-anti-mouse-HRP (Abcam ab6789, 1:1000). Blots were subsequently washed in TBST and ECL Prime Western Blotting Detection System (Amersham Biosciences) was applied to detect immunoreactivity. Chemiluminescent bands were detected using the Storm Scanner 860 (Amersham Biosciences). AR and KDM1A immunoreactive bands were confirmed by preabsorption of antibody with available blocking peptides for one hour at room temperature prior to overnight incubation.

**Table 1:** List of antibodies and their dilutions used for Western blot or IHC protocols.

Protein	Antibody for Western Blot and Dilution Utilized	Blocking Peptide Ratio to Antibody	Antibody for IHC and Dilution Utilized
AR	Santa Cruz sc816	Santa Cruz sc816-P	
	1:500	3:1	
KDM1A	Abcam ab17721	Abcam ab17763	Abcam ab17721
	1:500	2:1	1:100
KDM4D	Abcam ab93694		Abcam ab93694
	1:300		1:2000
VEGFA	Santa Cruz sc152		
	1:500		
DNMT1	Abcam ab92453		
	1:1000		
β-actin	Santa Cruz sc47778		
	1:1000		

Immunoreactive bands were quantified using ImageJ software (NIH). Pixelation for the protein of interest was divided by that of β-actin for each sample to normalize for protein loading. The average amount of normalized protein was then compared between control and TP treated ewe cotyledon and caruncle tissue using ANOVA followed by Tukey pairwise comparison (Minitab 16).

### Immunohistolocalization

To determine cellular localization of AR, KDM1A, and KDM4D proteins in the placenta, type A placentomes from control ewes (n = 4) and available first trimester human placenta (n = 3) were used for immunohistochemistry. Briefly, 5μm paraffin sections were cut from the center of the tissue blocks, placed on slides, deparaffinized with Citrosolve (Fisherbrand), and rehydrated with washes of decreasing percentages of ethanol. Slides were boiled for 15 minutes in 10mM sodium citrate pH6 for antigen retrieval using 5 minute intervals with 2 minute breaks. Non-specific peroxidase activity was inhibited by a 30 minute incubation in 3% hydrogen peroxide solution in PBS. Slides were incubated for 1 hour in 2% Superblock Blocking Buffer (Thermo Scientific, Waltham, MA) in PBS prior to incubation with KDM1A and KDM4D antibodies, and in 2% goat serum in PBS preceding all other antibodies. After blocking, slides were washed in PBS. Antibody dilutions are listed in Table 1. AR antibody was diluted in 2% goat serum in PBS. KDM1A and KDM4D antibodies were diluted in 2% Superblock Blocking Buffer in PBS. Primary antibodies were incubated overnight at 4°C. Control slides were incubated with 2% Superblock or 2% goat serum in PBS with exclusion of primary antibody. AR blocking peptide (Santa Cruz sc816-P) was used at a 3:1 ratio with antibody to confirm specific immunolocalization. After incubation with primary antibody, slides were washed in PBS and incubated with secondary antibody for 30 minutes at room temperature. A goat-anti-rabbit polyclonal secondary antibody tagged with HRP (Abcam ab6721, 1:1000) was used for AR, KDM1A, and KDM4D detection. Slides were washed in PBS and stained using avidin-biotin staining with diaminobenzidine (DAB) peroxidase substrate kit (Vector Labs, Burlingame, CA) to detect HRP immunoreactivity. Slides were washed again in PBS and dehydrated prior to mounting.

### Placentome RNA Isolation and Real Time PCR

Prior to real time PCR amplification, primer specificity was confirmed using DNA sequencing of PCR amplicons. Briefly, PCR analysis was performed using a placentome cDNA pool and GoTaq (Promega) per the manufacturer’s recommendations. PCR product was electrophoresed on a 1% agarose gel with a 100bp ladder (New England BioLabs) to determine the presence of expected amplicon sizes. The amplicon band was excised from the agarose gel and DNA was isolated using QIAquick Gel Extraction Kit (Qiagen). The resulting DNA was sequenced at the Colorado State University Proteomics and Genomics Laboratory. The sequences were blasted using NCBI blast to confirm specificity. Primer efficiency was assessed using serial dilutions of a cDNA pool from placentome samples. Standard curves from serial dilutions were analyzed on the Roche LightCycler 480 (Roche, Basel, Switzerland), and primer efficiencies used for real time PCR assay were calculated and are listed in S2 Table.

Total RNA was isolated from pulverized samples using the RNeasy Mini kit (Qiagen) and was treated with RNase-Free DNase (Qiagen) to eliminate any genomic DNA contamination. RNA quality and purity was determined with a NanoDrop 1000 Spectrophotometer (NanoDrop Technologies) and RNA aliquots were stored at -80°C. Total RNA was processed for reverse transcription using qScript (Quanta Biosciences). 1μg of total RNA was added to each reverse transcription reaction with 4μL reverse transcriptase QScript Supermix (Quanta Biosciences) and nuclease free water up to 20μL total reaction volume. Reverse transcription of total RNA was performed according to manufacturer’s specifications with 5 minutes 25°C, 30 minutes 42°C, 5 minutes 85°C, and holding at 4°C for use the same day. Resulting cDNA was used as a template for real time PCR quantification. cDNA was diluted 1:4 in nuclease free water prior to loading into real time PCR reactions. For real time PCR analysis, 2.5 μl of nanopure water was added to every 5μl of LightCycler 480 SYBR Green I Master (Roche, Basel Switzerland). Primer sets were added to reach a final concentration of 0.5μM in each reaction and 1μl of diluted cDNA was added for a final cDNA concentration of 1.25 ng/μL in a total volume of 10μL. Samples were loaded into 384 well LightCycler 480 plates (Roche) and analyzed using a LightCycler 480 PCR system (Roche Applied Science) in duplicate. Real time PCR cycle conditions were 95°C for 5 minutes, proceeded by 45 cycles of denaturing at 95°C for 30 seconds, annealing at 60°C for 15 seconds, and extension at 72°C for 10 seconds. Following real time PCR amplification, melt peaks were generated with an incubation at 95°C for 30 seconds to confirm a single amplicon was present. Cp values were normalized using the geometric mean of *RN18s* and *GAPDH*. Statistical analysis between TP treated and control samples ANOVA followed by Tukey pairwise comparison (Minitab 16). Results reported for real time PCR data use 2^-ΔCp^ values for statistical analysis and graphs, and fold changes between 2^-ΔCp^ values in tables for comparison [42].

### Coimmunoprecipitation of AR with KDM1A and KDM4D from Placentomes

Protein was isolated from pulverized snap frozen cotyledon and caruncle tissue from control and TP treated ewes in RIPA buffer containing 10mM Tris-HCl pH 7.5, 140mM NaCl, 1mM EDTA, 0.5mM EGTA, 1% Triton X-100, 0.1% SDS, 0.1% proteinase inhibitor, and 0.1% 100mM PMSF. Protein concentration was determined by a BCA assay (Pierce). 250 μg of cotyledon and 250 μg caruncle protein were diluted in RIPA buffer to reach a final concentration of 1 μg/μL in 500 μL RIPA buffer. Antibodies were combined with 500 μL of diluted placentome protein and incubated for an hour at 4°C on a rotational mixer. 2 μg of AR antibody (Santa Cruz, sc816), 2 μg of KDM1A antibody (Abcam, ab17721), 2 μg KDM4D antibody (Abcam, ab93694), or 1 μg secondary antibody as a negative control (Abcam, anti-rabbit ab6721) were added to each sample. Preabsorption of AR and KDM1A antibodies with their blocking peptides (4 μg) occurred at room temperature 1 hour prior to incubation with placentome protein. Following antibody incubation, 20 μL of A/G PLUS agarose beads (Santa Cruz, sc2003) were added for an hour at 4°C on a rotational mixer. Samples were briefly spun at 2500 rpm for 5 minutes at 4°C. Agarose beads were centrifuged and washed two times with RIPA buffer followed by three washes with PBS. Protein was resuspended in 6.67 μL 6x Western blot loading dye (described above) diluted with 31.33 μL RIPA buffer, then stored at -80°C overnight. The following day, samples were thawed and 2 μL of β-mercaptoethanol was added to reach a final volume of 40 μL. Samples were immediately incubated at 95°C for 12 minutes and centrifuged at 10,000 x g for 1 minute at room temperature. 20 μL of immunoprecipitated protein was loaded per lane on 10% Tris-HCl gels (BioRad) and electrophoresed. Western blot protocol and antibody dilutions followed those described above and in Table 1.

### Chromatin Immunoprecipitation with AR and KDM1A in Placentomes

ChIP was performed using ChIP-It Express High Throughput (Actif Motif) following the manufacturer’s protocol. Approximately 2 mg of pulverized cotyledon and caruncle tissue from control and TP treated ewes was added to 500 μL of ice cold PBS. Cotyledon and caruncle tissue was mixed for each ewe and mechanically homogenized by pestle on ice. 14 μL of 37% formaldehyde (VWR International) was added, samples were vortexed, then incubated on ice for 8 minutes. 57 μL of 125 mM glycine was added to quench crosslinking. Samples were vortexed and incubated on ice for 5 minutes. Samples were centrifuged for 2 minutes at 10,000 x g at 4°C. Supernatant was aspirated off and samples were resuspended in 500 μL ice cold PBS. Samples were centrifuged again, supernatant was removed, and samples were resuspended in 500 μL PBS. 130 μL of lysis buffer containing 50mM Tris HCl pH8.0, 10mM EDTA, 1% SDS, and 1:100 proteinase inhibitor was added. Samples were homogenized by pestle, vortexed, and incubated on ice for 30 minutes. Samples were homogenized again then sonicated using a Biorupter for 30 minutes with cycles of 5 seconds on, 5 seconds off. 400 μL of RIPA buffer was added, then samples were centrifuged for 10 minutes at 12,000 x g at 4°C. Supernatant was placed in a new tube and remaining sample was resuspended again in 400 μL RIPA buffer and centrifuged. Supernatant was combined and DNA concentration was determined by Nanodrop 1000 Spectrophotometer (NanoDrop Technologies). 6.3 μg of DNA was placed in a new tube for immunoprecipitation.

25 μL of magnetic G-protein beads (Active Motif) were added with 10 μL of ChIP buffer 1 (Actif Motif) and 1 μL of proteinase inhibitor. 2 μg of AR antibody (Santa Cruz, sc816), 2 μg of KDM1A antibody (Abcam, ab17721), or 2 μg anti-rabbit secondary antibody (Abcam ab6721, negative control) were added to each sample. RIPA buffer was added to bring the total immunoprecipitation reaction volume up to 200 μL. Samples were incubated overnight at 4°C on a rotational mixer, and briefly spun at 2500 rpm for 1 minute at 4°C. Magnetic G-protein beads were pulled down and washed two times with 200 uL of ChIP buffer 1, then washed 3 times with 100 μL of ChIP buffer 2 (Actif Motif). Magnetic beads were then resuspended in elution buffer AM2 and incubated at room temperature for 15 minutes on a rotational rotor. Samples were centrifuged at 2500 rpm for 1 minute at room temperature. 50 μL of reverse crosslink buffer was added and samples were vortexed. Magnetic beads were pulled down and supernatant was removed and placed in a new tube. 10 μL of input cross-linked DNA was added to a tube containing 88 μL of ChIP buffer 2 and 2 μL of 5 M NaCl as a PCR positive control (ChIP input DNA). All samples were incubated overnight at 64°C. Samples were cooled to room temperature and 2 μL of proteinase K was added. Samples were incubated for 1 hour at 37°C, then 2 μL of Proteinase Stop Solution was added.

1 μL of ChIP sample was added per each PCR reaction in technical triplicates. Sonicated DNA samples and ChIP input DNA were used as positive controls for PCR. PCR and amplicon sequencing was performed as described above. Androgen response element (ARE) half-sites were identified in a-5000bp region of the 5’ flanking sequence of bovine and human *VEGFA* as the promoter region of ovine *VEGFA* has not been published. Primers were designed to amplify an ARE region of genomic DNA situated between two ARE half-site in bovine *VEGFA*, 181 nucleotides away from the upstream ARE and 44 nucleotides away from the downstream ARE, and was located 3,118 nucleotides away from the coding region. Primers also were designed to span a region 1,774 nucleotides from an ARE half-sites (non-ARE) in the-5,000 5’ flanking sequence in the human *VEGFA*, located 4,786 nucleotides away the coding region (primers listed in Table 2).

**Table 2:** List of primer sequences used for PCR of ChIP samples spanning an ARE and non-ARE region in the-5000bp 5’UTR of *VEGFA*.

Gene	ARE/non-ARE Region	Primer Sequence	Amplicon Size
*VEGFA*	ARE	F-5′ CTCTGTCTGGGCTGCTCTCT	179
		R-5′ TCCTCCCATGGACGTAACTC	
*VEGFA*	Non-ARE	F-5′ GCCTGTAATCCCAGCACTCT	181
		R-5′ GAGCAATTCTCCTGCCTCAG	

## Results

### Placentome Morphology and Fetal Growth

**Fig 1:**
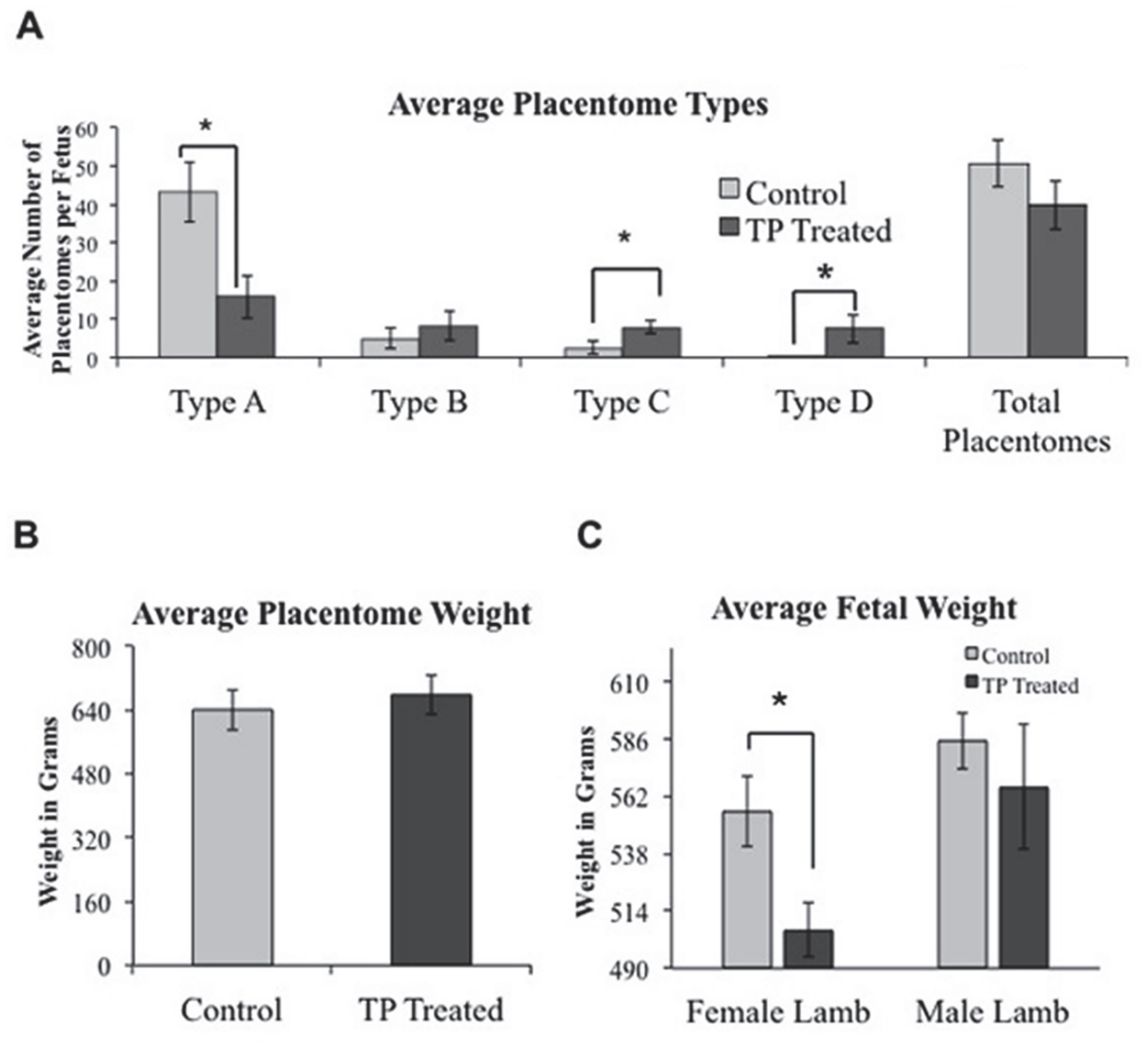
Placentome morphology and fetal weight. **A**) Testosterone propionate treatment (TP) decreased the number of type A placentomes, and increased type C and type D placentomes collected at gestational day 90. **B**) TP treatment did not affect placental weight while **C**) female fetuses from TP ewes had significantly reduced body weight at gestational day 90 compared to female fetuses from controls. * Indicates P≤0.06

The total number and weight of placentomes collected from control and TP treated ewes did not differ at GD 90 (Fig. 1A and 1B). Placentas from TP treated ewes had a reduced number of type A placentomes (P = 0.001) and an increased number of type C (P = 0.002) and type D (P = 0.06) placentomes compared to control ewes (Fig. 1A). There was no difference between weight of male (n = 4) and female (n = 7) fetuses in control pregnancies or between male (n = 4) and female (n = 4) fetuses from TP treated ewes. However, female fetuses from TP treated ewes weighed less (P = 0.022) compared to female fetuses from control ewes (Fig. 1C).

### Changes in Epigenetic Factors and Steroid Hormone Receptors

**Fig 2:**
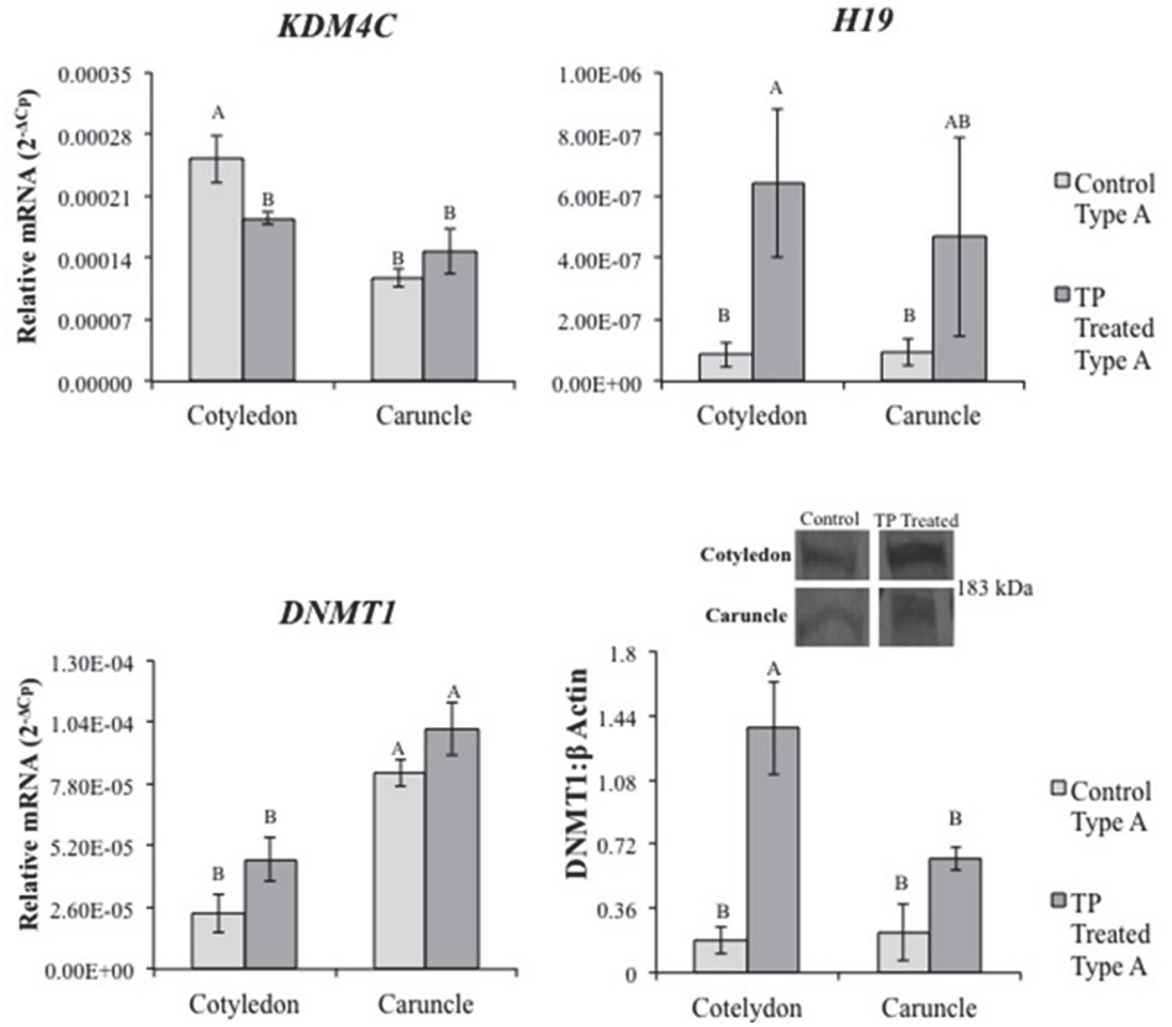
Differential levels of epigenetic regulators with TP treatment. Analysis of relative mRNA levels for genes regulating histone and DNA methylation and imprinting in ovine placentome tissue from real time PCR. *KDM4C* was decreased in type A placentomes from TP treated ewes compared to controls. *H19* was increased in cotyledon tissue from type A placentomes in TP treated ewes compared to controls. *DNMT1* increased in TP treated ewe type A cotyledon tissue compared to controls. DNMT1 increased in TP treated ewe type A placentomes (P<0.001). Different letters indicate statistical difference of P<0.05.

**Fig 3:**
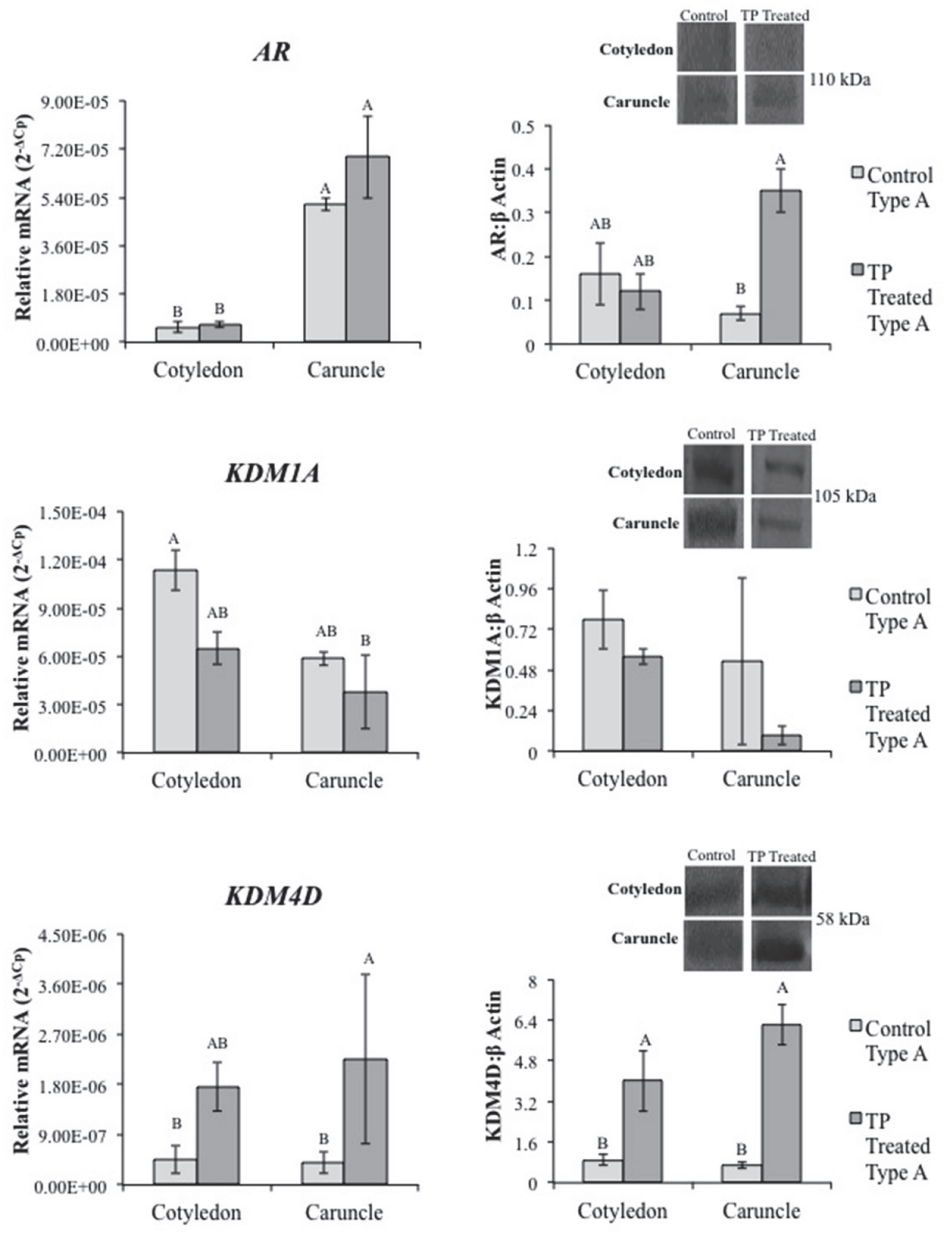
Real time PCR and representative Western blot depicting placentome mRNA and protein levels of AR, KDM1A and KDM4D in control and TP treated ewes. TP treatment did not alter mRNA for *AR*, *KDM1A*, or *KDM4D*. AR was increased in type A caruncle tissue from TP treated ewes compared to controls. No difference was found in KDM1A, though KDM4D increased in type A placentomes from TP treated ewes compared to controls. Placentome values represent average of cotyledon and caruncle quantified mRNA levels in type A cotyledons. Different letters indicate statistical difference of P<0.05.

In addition to altered gross morphology of the placentomes, TP treatment led to global changes in DNA methylation (S1 Fig.) indicating prenatal androgenization is associated with epigenetic changes. Transcript levels for several genes involved in epigenetics (*KDM’*s, *DNA methyltransferases* (*DNMT*s), and imprinted long non-coding *H19*) were assessed in GD90 placentomes from control and TP treated ewes (Figs. 2, 3, and S2 Fig.). While no difference was observed in *KDM3A* or *KDM4A* mRNA levels (S1 Fig.), *KDM4C* was lower in type A cotyledons from TP treated ewes compared to controls (P<0.001, Fig. 2). *H19* mRNA levels were greater in cotyledon tissue from type A placentomes from TP treated ewes compared to controls (P = 0.005, Fig. 2). *DNMT1* levels were greater in type A cotyledon tissue from TP treated ewes compared to controls (P<0.001, Fig. 2). More DNMT1 protein also was observed in TP treated ewe type A cotyledon and placentome tissue (P<0.001, Fig. 2). No difference was observed in *DNMT3a* or *DNMT3b* with TP treatment (S2 Fig.). TP treatment did not alter placentome *AR*, *KDM1A*, and *KDM4D* mRNA levels (Fig. 3).

Further characterization of AR, KDM1A and KDM4D in GD90 ovine placentomes was completed to determine their response to TP treatment (Fig. 3). AR protein was greater in caruncle tissue from type A placentomes in TP treated ewes compared to controls (P = 0.014) (Fig. 3). Preabsorption with the complementary AR peptide resulted in loss of immunoreactive band (S3 Fig.). No significant difference was observed in KDM1A protein. Although TP treatment did not alter *KDM4D* mRNA levels, KDM4D protein levels were greater in type A placentome tissue from TP treated ewes when compared to controls (P<0.001), but was not different in type D placentomes from TP treated ewes (Fig. 3).

### Localization of AR, KDM1A and KDM4D in GD90 Placentomes

**Fig 4:**
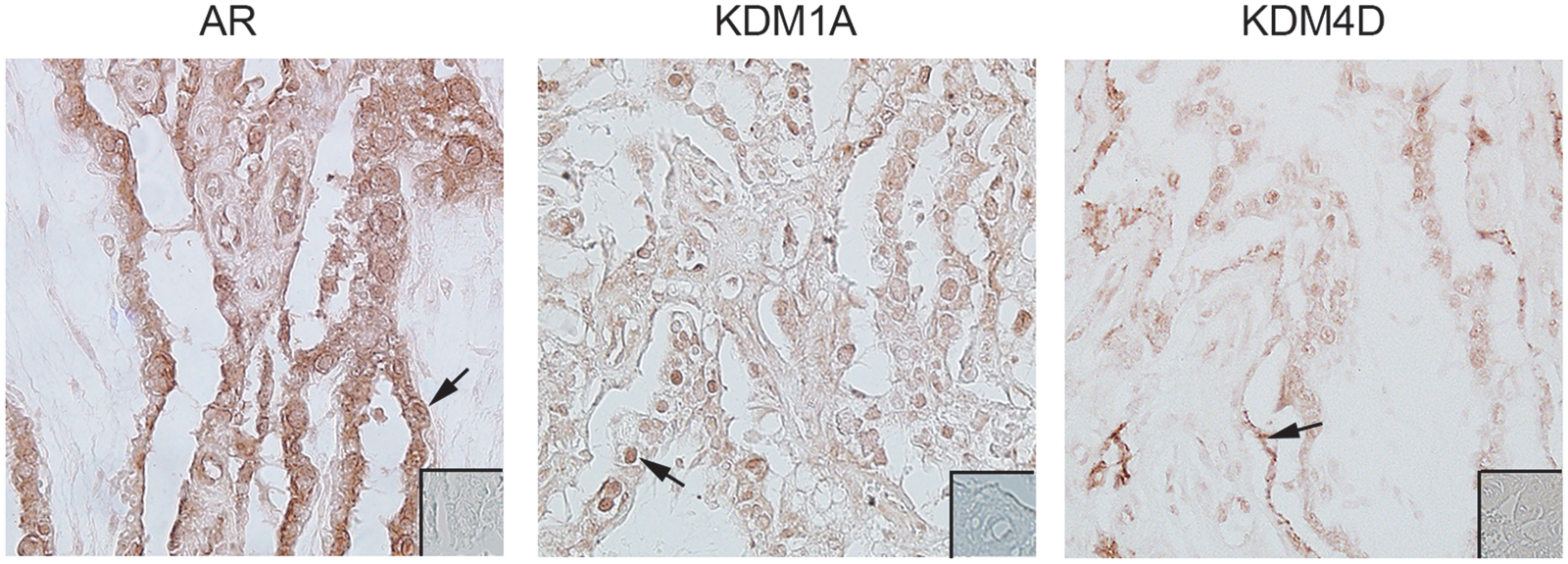
Immunolocalization of AR, KDM1A, and KDM4D in type A placentomes from control ewes at GD90. Inserts represent control IHC straining. Immunolocalization of AR was present in the trophoblasts of the villous epithelium with primarily nuclear staining (arrow). KDM1A immunolocalized to the nucleus of the trophoblasts in the villous epithelium (arrow), while KDM4D immunostaining was prominent in the apical surface of maternal uterine epithelium (arrow). Images were taken at 20X maginification.

In type A placentomes from control ewes, nuclear AR immunolocalized to the trophoblast and syncytium (Fig. 4). KDM1A was detected in nuclei of trophoblast cells, and AR and KDM4D both immunolocalized to the syncytium. Predominate immunostaining for KDM1A was observed in trophoblast nuclei, while KDM4D localized to the apical surface of the syncytium (Fig. 4).

### mRNA Levels of Androgen Responsive Genes in Placentomes

**Fig 5:**
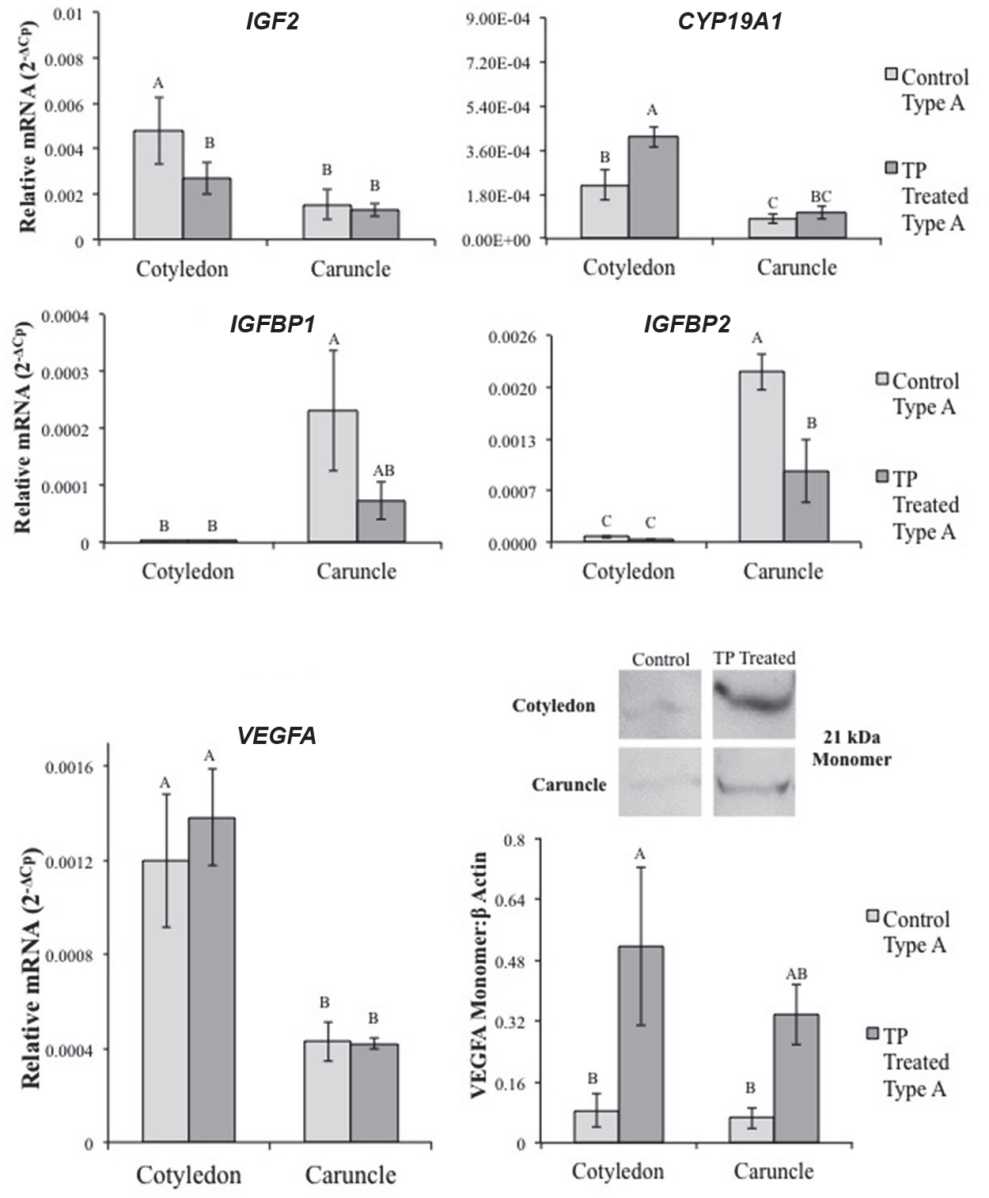
Real time PCR results of AR target-genes known to regulate trophoblast differentiation and proliferation. *IGF2* increased in type D placentomes from TP treated ewes compared to type A placentomes from control or TP treated ewes. *IGFBP1* and *IGFBP2* both decreased in caruncle tissue from type A placentomes from TP treated ewes compared to controls. *IGFBP2* also decreased in type A and D placentomes from TP treated ewes compared to control type A placentomes. *CYP19* increased in type D placentomes form TP treated ewes compared to type A placentomes from controls. TP treated ewes had increased *VEGFA* in type D placentomes compared to type A placentomes from control ewe. TP treated ewes had increased VEGFA monomer in type A placentome cotyledon tissue. Different letters indicate statistical difference of P<0.05.

Real time PCR of selected androgen responsive genes (Androgen Responsive Gene Database; http://argdb.fudan.edu.cn/) known to regulate placentation was undertaken to determine if TP treatment altered relative mRNA levels (Fig. 5 and S4 Fig.). *Insulin-like growth factor 2* (*IGF2*) was lower in cotyledon tissue from type A placentomes from TP treated ewes compared to controls (P<0.001, Fig. 5). *IGF binding protein 2 (IGFBP2)* was lower in caruncle tissue from TP treated ewes compared to type A placentomes from control ewes (P<0.001). *Aromatase (CYP19A1)* mRNA levels were greater in type A placentomes from TP treated ewes compared to type A placentomes from controls (P<0.001, Fig. 5). *Vascular endothelial growth factor* (*VEGFA*) was greater in cotyledon compared to caruncle tissue in type A placentomes from control as well as TP treated ewes (P<0.001, Fig. 5), and was not different between control and TP treated tissues. However, VEGFA protein monomer was higher in cotyledon tissue in type A placentomes from TP treated ewes compared to controls (P = 0.005, Fig. 5).

### AR Complexes with Histone Demethylases and binds to VEGFA

**Fig 6:**
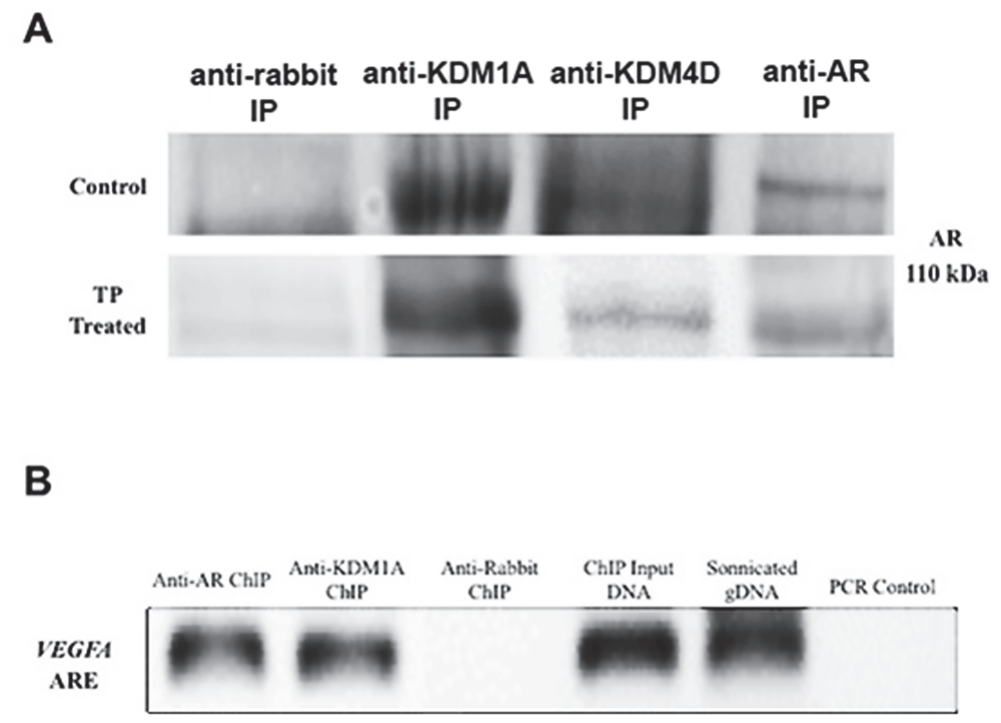
Interaction of KDMs with AR. **A**) Immunoprecipitation of AR, KDM1A, and KDM4D from placentome protein isolated from control and TP treated ewes followed by Western blot detection of co-immunoprecipitated protein. Anti-rabbit IP represents pull down by secondary antibody used for Western blot immunolabeling. Antibody preabsorption with blocking peptide was used as a negative control. **B**) PCR of an ARE promoter region of *VEGFA* from ChIP samples. IP, immunoprecipitate; ChIP, chromatin immunoprecipitation; ARE, primers spanning androgen response element in promoter region

Coimmunoprecipitation and chromatin immunoprecipitation were used to determine if AR interacts with KDM1A and/or KDM4D in ovine placenta to regulate androgen responsive *VEGFA*. Co-immunoprecipitation and Western blot revealed AR binds to KDM1A and KDM4D in GD90 placentomes (Fig. 6A, S5 Fig.), and preabsorption with AR or KDM1A blocking peptide decreased or inhibited immunoreactive bands, respectively (S6 Fig.). Furthermore, both AR and KDM1A protein bind the same DNA region in the 5’ flanking sequencing of *VEGFA* containing an ARE half-site (Fig. 6B) according to PCR amplification. PCR with primers designed for a non-ARE region in the 5’ flanking sequencing of *VEGFA* did not amplify in AR or KDM1A chromatin immunoprecipitation samples, but was present in genomic DNA (data not shown).

### Immunolocalization of KDM1A, and KDM4D in First Trimester Human Placenta

**Fig 7:**
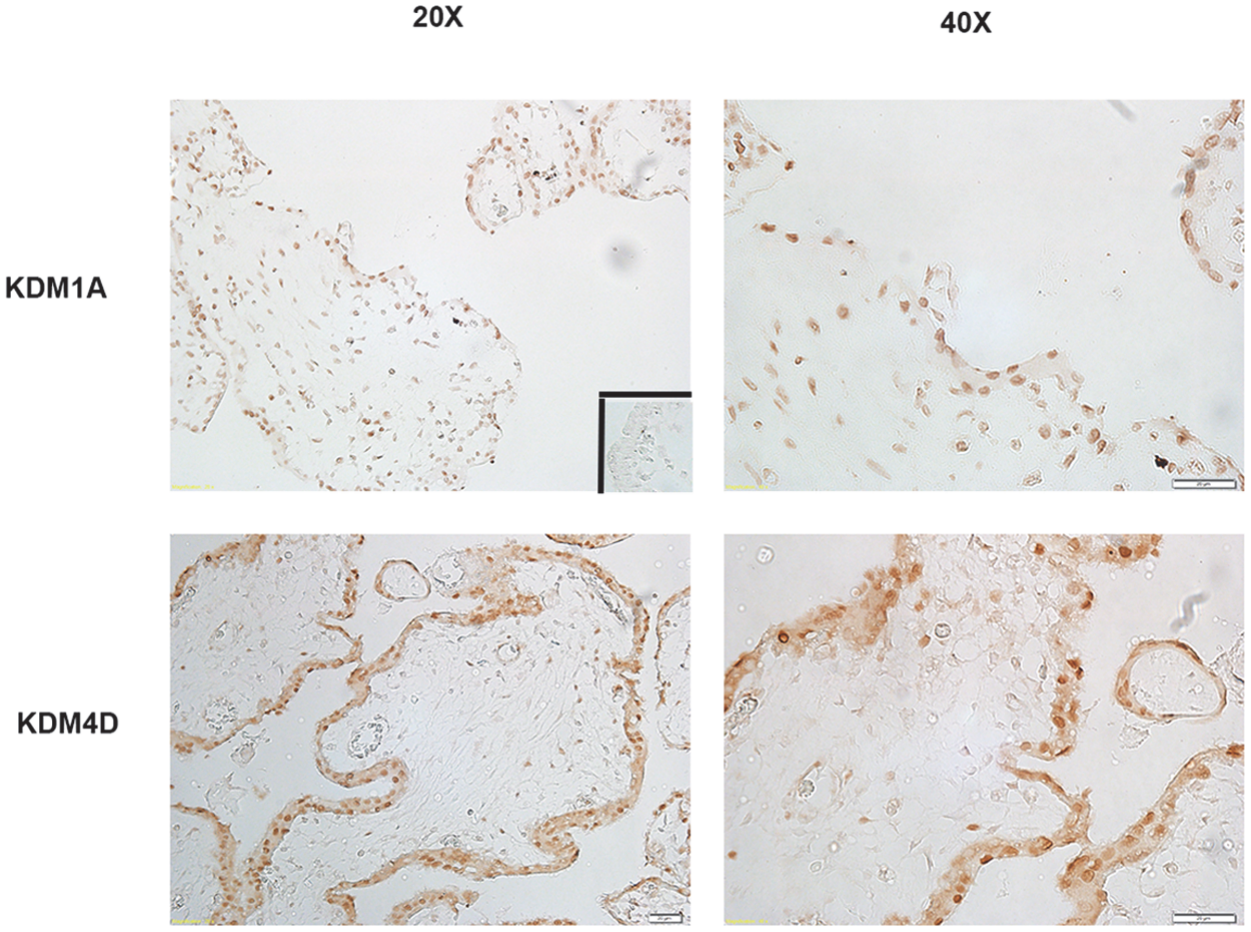
Immunolocalization of KDM1A and KDM4D in first trimester human placenta samples. KDM1A immunostaining also localized to the nuclei in the syncytium and cells in the villous stroma at 11.5 weeks of gestation. KDM4D immunolocalization was similar to KDM1A, with nuclear immunostaining present in the syncytium and in the villous stroma at 11.5 weeks of gestation. White scale bar = 20μm for 20X and 40X. Insert is a representative image from control slides.

In 11.5 weeks of gestation tissue samples, KDM1A, and KDM4D immunolocalized to nuclei in syncytiotrophoblast (Fig. 7). Additional immunostaining was observed in cells in the villous stroma at 11.5 weeks of gestation.

## Discussion

Previous studies revealed that the placenta contains AR, and normal pregnancy is associated with an increase in serum testosterone levels [11,12]. Moreover, abnormal testosterone levels are associated with a number of placental-associated disorders, including preeclampsia and intrauterine growth restriction, whereas conditions such as polycystic ovarian syndrome lead to pregnancy complications [13,14]. Prenatal androgenization models previously have been employed to study abnormal developmental programming, and the prenatal androgenized ewe has demonstrated to be an excellent model, especially for PCOS in women [36,38,39]. For example, adult ewes that were exposed to increased androgens during the first half of gestation in this model exhibit hyperandrogenism, anovulation, metabolic dysfunction, and infertility. However, no study to date has described the presence of AR in sheep placentomes. In our study, prenatal androgenization from GD30 to 90 led to reduced fetal weight of female lambs. Androgen induced intrauterine growth restriction has been described in sheep [36,38] and prenatal androgenized rats [43] along with reduced placental weights [44]. These data suggest androgen as a regulator of fetal growth and placental function. We also report that prenatal androgenization alters placentome morphology with the appearance of greater numbers of type D placentomes. While the increase in type C and D placentomes has been associated previously with prenatal androgenization [45], increased presence of type C and D placentomes in pregnancies complicated by maternal nutrient restriction corresponds to increased interdigitated fetal villi, heightened cotyledon proliferation and increased vascular density [45–48].

In addition to altered ovine placentome morphology and decreased global DNA methylation in placentomes from TP treated ewes, we speculated abnormal epigenetic regulation of trophoblast gene expression was induced. Proper regulation of DNA methylation in trophoblasts is necessary for normal placenta developmental programming. For example, reduction of DNA methyltransferases (DNMT1, DNMT3a and DNMT3b) in choriocarcinoma (BeWo) cells prevents cell migration [49], and reduced placental DNMT1 expression in *in vitro* produced ovine embryos is associated with early embryonic loss [50]. Similarly, loss of DNA methylation imprinting in long non-coding *H19* and neighboring *IGF2* in the placenta is associated with Beckwith-Wiedemann Syndrome and increased prenatal and placental growth [51,52]. In the prenatal androgenization ewe model, we report up-regulated *H19* and *IGF2* in placentomes from TP treated ewes, suggesting that excess prenatal androgen exposure may lead to dysregulated imprinting in the placenta via decreased global DNA methylation, contributing to abnormal fetal growth observed with prenatal androgenization [36,37,43,44].

Regulation of gene expression involves histone modifications with site-specific adaptations of amino acids in histone N-terminal tails, including methylation of lysine residues [53,54]. KDMs demethylate lysines in histones to typically increase DNA accessibility and promote transcription in the presence of transcription activators, such as ligand-bound nuclear receptors. Previous studies in cancer cells demonstrate that KDMs interact with and bind to sex hormone receptors to regulate androgen and estrogen signaling [32,35]. In addition, knock-out of *KDM1A* in mice results in early embryonic lethality [55,56]. Considering the many similarities between cancer and placental cells, we reasoned that KDM’s also are present and involved in mediating androgen signaling in placental cells. Not only are AR and histone demethylases KDM1A and KDM4D present in ovine placenta and first trimester human placenta, but AR co-localizes and complexes with KDM1A and KDM4D. This agrees with previous studies that reported AR heterocomplexes with KDMs to regulate target genes [27–35], though this is the first study to our knowledge to show this interaction also occurs in placental tissue.

With the decreased DNA methylation observed in placental tissue from TP treated ewes, the shift in placentome morphology may be a direct result of altered gene expression, more specifically from increased growth and angiogenic factors induced by androgen signaling in placental tissues. In GD90 placentomes from TP treated ewes, we observed increased mRNA levels of AR target genes *insulin-like growth factor 2 (IGF2)* and *vascular endothelial growth factor (VEGF)*, and increased protein levels of AR and VEGFA. During prenatal development, IGFs and VEGFA regulate placentation and fetal growth. IGFs are expressed throughout the placenta of various species, including maternal decidualized cells, cytotrophoblasts, and chorionic mesoderm [57,58] and function as a key regulator of prenatal growth [59]. Decreased *IGFBP1* and *IGFBP2* in caruncle tissue from TP treated ewes may be an additional mechanism to heighten local IGF signaling in androgenized placenta [59], explaining the rise in placentome overgrowth observed with the increase in type C and D placentomes [46–48].

VEGFA is a critical regulator of placental development and function and stimulates fetal and placental growth through increased angiogenesis [60,61]. While androgen responsive VEGFA stimulates placental and prenatal angiogenesis, it also stimulates differentiation of extravillous trophoblasts that remodel maternal spiral arteries in the human decidua for enhanced blood flow to the placenta [60]. Insufficient extravillous trophoblast differentiation and invasion has been associated with placental insufficiency and the development of pregnancy induced pathologies, including fetal growth restriction and preeclampsia [62]. Multiple studies have also shown that maternal plasma testosterone is increased in cases of severe preeclampsia [63,64], with placentas from preeclamptic pregnancies expressing increased syncytiotrophoblast and stromal cell expression of AR [19]. Mutations in *AR* are correlated with increased risk for the development of preeclampsia [65]. As we demonstrated AR and KDM1A binding to an ARE in *VEGFA*’s promoter region in ovine placental tissue, placental androgens may have an important function in regulating trophoblast function in sheep, possibly through VEGFA signaling. Although KDM4D also complexed with AR, the KDM4D antibody was not suitable for ChIP and as such we were not able to demonstrate if KDM4D possibly is involved with AR regulation of VEGFA in placentomes.

This proposed mechanism for placental androgen signaling is further supported by evidence that hypoxia induces AR activity and expression of androgen-regulated genes, including VEGFA [24–26], while androgen withdrawal leads to hypoxia [66]. Although nothing is known about the role of KDMs in placental cells, KDMs are regulated by hypoxia to promote vascularization, invasion, migration, and cell proliferation in cancer tissue [27,34,35,67]. This is of particular interest as hypoxia-regulated trophoblast differentiation and invasion in the first trimester is required to establish proper placentation [1,62,68].

Prenatal androgenization and increased ligand could promote AR-KDM1A complex recruitment to AR-target genes in the placenta to stimulate gene transcription by promoting reduced methylation signatures near target genes [28,53]. Therefore, ligand-dependent recruitment of AR-KDM1A to androgen target genes could regulate growth, invasion, and angiogenesis and, as such, placental development and function. However, despite the observed up-regulation in mRNA levels for AR-target genes regulating growth, invasion, and angiogenesis, GD90 prenatal androgenized ewe lambs had reduced fetal weight, suggesting that increased androgen signaling, either directly or indirectly, dysregulated fetal developmental programming through other factors, such as placental nutrient transport, that were not investigated.

In conclusion, the present study revealed that prenatal androgenization alters ovine placentome development and is associated with decreased global DNA methylation and altered gene transcription of angiogenic and growth factor pathways. Importantly, we demonstrated for the first time that AR complexes to KDM’s in ovine placenta, and that AR and KDM1A bind to the same promoter region of AR-target gene *VEGFA* in trophoblast cells. These results indicate androgen signaling functions jointly with KDMs to play a regulatory role in placental development. Finally, we were able to detect KDM1A and KDM4D in syncytiotrophoblast and in villous stromal cells in first trimester human placenta, indicating AR-KDM signaling also has a possible role in human trophoblast differentiation. Current studies are underway to determine the role of histone demethylases in human placentation and to determine androgen’s effects on trophoblast differentiation.

## Supporting Information

S1 Fig Global methylation in ovine GD90 placentomes. Global DNA methylation decreased in type A placentomes from TP treated ewes when compared to type A placentomes from controls. * Indicates P<0.05.
(TIF)

S2 Fig Real time PCR results for other selected epigenetic regulators. Difference in letter indicates significant difference of P<0.05.
(JPG)

S3 Fig Representative Western blot blocking peptide controls. Loss of immunoreactive band for AR when antibody is preabsorbed with AR blocking peptide at a 1:3 ratio. LNCaP, human prostate adenocarcinoma cells.
(JPG)

S4 Fig Real time PCR results for other AR target-genes known to regulate trophoblast differentiation and proliferation. Placentome values represent average of cotyledon and caruncle quantified mRNA levels in type A cotyledons. Difference in letter indicates significant difference of P<0.05.
(JPG)

S5 Fig Interaction of KDM1A with AR. Western blot detection of KDM1A (105 kDa) from placentome protein following immunoprecipitation (IP) using AR and KDM1A antibodies. Anti-rabbit IP represents pull down by secondary antibody as a negative control.
(TIF)

S6 Fig Reduced AR immunoreactive band in immunoprecipitation of AR when antibody is preabsorbed with blocking peptide (1:1 dilution). Loss of KDM1A immunoreactive band in immunoprecipitation when antibody is preabsorbed with blocking peptide (1:1 dilution). IP = immunoprecipitation.
(JPG)

S1 Table List of ewes, number of fetuses, fetal sex and placentome number per pregnancy. (DOC)

S2 Table Primer sequences used for real time PCR analysis of sheep placentomes. (DOC)
