# Supplementary figures and images for: Androgen Receptor and Histone Lysine Demethylases in Ovine Placenta
Source: PLoS One. 2015 Feb 12;10(2):e0117472. doi: 10.1371/journal.pone.0117472 (PMC4326353; doi:10.1371/journal.pone.0117472)

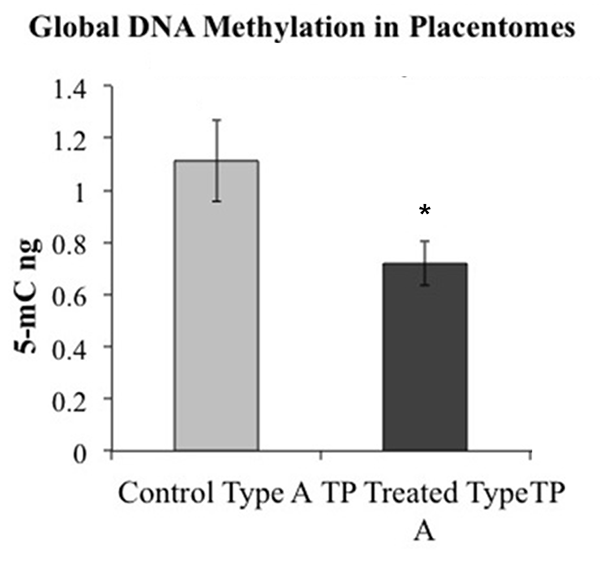

Supplement: S1 Fig — Global DNA methylation decreased in type A placentomes from TP treated ewes when compared to type A placentomes from controls. * Indicates P<0.05. (TIF) [file pone.0117472.s001.tif]

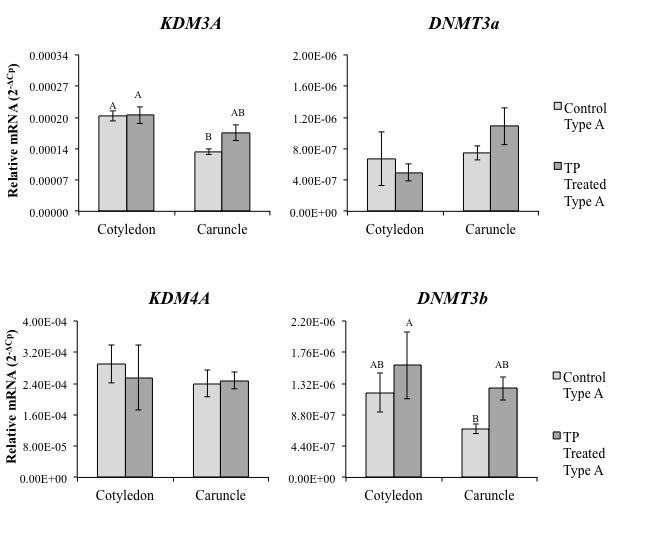

Supplement: S2 Fig — Difference in letter indicates significant difference of P<0.05. (JPG) [file pone.0117472.s002.jpg]

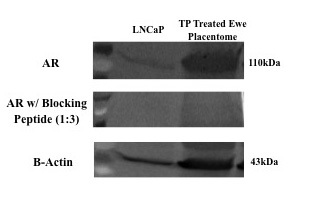

Supplement: S3 Fig — Loss of immunoreactive band for AR when antibody is preabsorbed with AR blocking peptide at a 1:3 ratio. LNCaP, human prostate adenocarcinoma cells. (JPG) [file pone.0117472.s003.jpg]

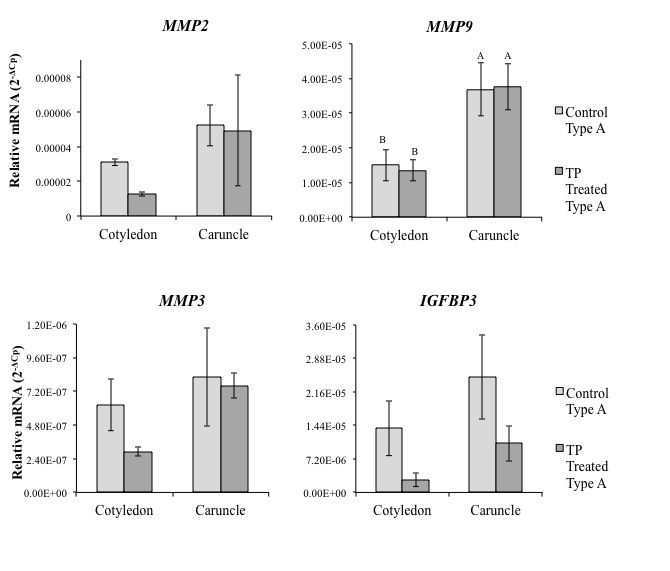

Supplement: S4 Fig — Placentome values represent average of cotyledon and caruncle quantified mRNA levels in type A cotyledons. Difference in letter indicates significant difference of P<0.05. (JPG) [file pone.0117472.s004.jpg]

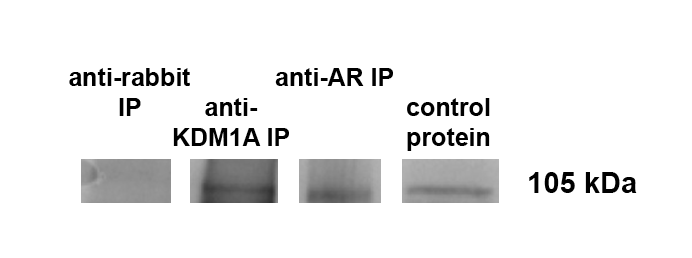

Supplement: S5 Fig — Western blot detection of KDM1A (105 kDa) from placentome protein following immunoprecipitation (IP) using AR and KDM1A antibodies. Anti-rabbit IP represents pull down by secondary antibody as a negative control. (TIF) [file pone.0117472.s005.tif]

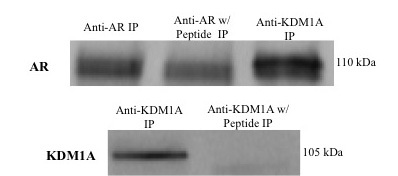

Supplement: S6 Fig — Loss of KDM1A immunoreactive band in immunoprecipitation when antibody is preabsorbed with blocking peptide (1:1 dilution). IP = immunoprecipitation. (JPG) [file pone.0117472.s006.jpg]
