# Supplementary material for: Androgen Receptor and Histone Lysine Demethylases in Ovine Placenta
Source: PLoS One. 2015 Feb 12;10(2):e0117472. doi: 10.1371/journal.pone.0117472 (PMC4326353; doi:10.1371/journal.pone.0117472)
Supplement: S1 Table — (DOC) [file pone.0117472.s007.doc]

|  | **Ewe ID** | **Pregnancy Status** | **Fetal Sex** | **Placentome Number** |
| --- | --- | --- | --- | --- |
| **CONTROL** | 101 | Singleton | F | 37 |
|  | 114 | Singleton | F | 78 |
|  | 120 | Twins | F | 28 |
|  |  |  | F | 45 |
|  | 121 | Twins | F | 38 |
|  |  |  | F | 45 |
|  | 144 | Twins | F | 55 |
|  |  |  | M | 26 |
|  | 103 | Singleton | M | 94 |
|  | 137 | Twins | M | 53 |
|  |  |  | M | 58 |
| **TP TREATED** | 113 | Singleton | F | 63 |
|  | 131 | Singleton | F | 27 |
|  | 118 | Twins | F | 30 |
|  |  |  | M | 49 |
|  | 107 | Twins | F | 27 |
|  |  |  | M | 33 |
|  | 167 | Singleton | M | 29 |
|  | 138 | Singleton | M | 55 |

SUPPLEMENTAL TABLE 1. List of ewes, number of fetuses, fetal sex and placentome number per pregnancy.
