# Supplementary material for: Androgen Receptor and Histone Lysine Demethylases in Ovine Placenta
Source: PLoS One. 2015 Feb 12;10(2):e0117472. doi: 10.1371/journal.pone.0117472 (PMC4326353; doi:10.1371/journal.pone.0117472)
Supplement: S2 Table — (DOC) [file pone.0117472.s008.doc]

| **Gene** | **Primer Sequence** | **Amplicon Size** | **Primer Efficiency** | | |
| --- | --- | --- | --- | --- | --- |
| ***AR*** | **F-5' TCCTGGATGGGGCTTATGGT** | **150** | **99.8%** | | |
|  | **R-5' GCCTCATTCGGACACACTGG** |  |  | | |
| ***CYP19*** | **F-5' GTTGTGCCTATTGCCAGCAT** | **137** | **88.7%** | | |
|  | **R-5' AACCTGCAGTGGGAAATGAG** |  |  |  | |
| ***DNMT1*** | **F-5' TTCTGCAGCAAGAAGAGCAA** | **229** | **99.2%** | | |
|  | **R-5' AGAAGTCCTGGAGGCACTCA** |  |  | | |
| ***DNMT3A*** | **F-5' AGCACAACGGAGAAGCCTAA** | **180** | **96.3%** | | |
|  | **R-5' GTTCTTGCAGTTTTGGCACA** |  |  | | |
| ***DNMT3B*** | **F-5' TGCAGACAGCACCGAGTATC** | **190** | **90.1%** | | |
|  | **R-5' CTGCTGGAATCTCGGAGAAC** |  |  | | |
| ***H19*** | **F-5' AATAGAAGCCCCCTGGGTGT** | **152** | **83.1%** | | |
|  | **R-5' CCCCATCAGATCCCTGTCAT** |  |  | | |
| ***KDM1A*** | **F-5' ACATTGCAGTTGTGGTTGGA** | **216** | **99.1%** | | |
|  | **R-5' GACCCCAGAGCCTATGATGA** |  |  |  | |
| ***KDM3A*** | **F-5' GCCAACATTGGAGACCACTT** | **230** | **95.6%** | | |
|  | **R-5' GCACCTTGTTGGCAGTTTTT** |  |  |  | |
| ***KDM4A*** | **F-5' TGGATCGAGTATGGCAAACA** | **201** | **86.0%** | | |
|  | **R-5' TCTCAGGGCCAGTTCACTCT** |  |  |  | |
| ***KDM4C*** | **F-5' TGCACTTGCAGGAAAGAC** | **206** | **98.1%** | | |
|  | **R-5' ACACTGGAAGCTCCTGGATG** |  |  |  | |
| ***KDM4D*** | **F-5' GGCAGAGTACCGCCACTTAG** | **167** | **86.3%** | | |
|  | **R-5' GGTTCCACTGCTTCGTGTTT** |  |  |  | |
| ***MMP2*** | **F-5' ACCAGAGCACCATTGAGACC** | **220** | **86.5%** | | |
|  | **R-5' TGGATCCGAGAAAACCGTAG** |  |  |  | |
| ***MMP3*** | **F-5' GCAAGCAGGTTACCCAAGAG** | **156** | **99.5%** | | |
|  | **R-5' GGCTCCATGGATTGTGTCTT** |  |  | | |
| ***MMP9*** | **F-5' CTCCTACTCCTCCTGCACCA** | **205** | **99.3%** | | |
|  | **R-5' GCGTCCATCGGAGGTACA** |  |  | | |
| ***VEGF*** | **F-5' TCACCAAAGCCAGCACATAG** | **179** | **97.9%** | | |
|  | **R-5' GCGAGTCTGTGTTTTTGCAG** |  |  |  | |
| ***IGF2*** | **F-5' GACCGCGGCTTCTACTTCAG** | **202** | **98.6%** | | |
|  | **R-5' AAGAACTTGCCCACGGGGTAT** |  |  |  | |
| ***IGFBP1*** | **F-5' TGATGACCGAGTCCAGTGAG** | **248** | **94.5%** | | |
|  | **R-5' GTCCAGCGAAGTCTCACAC** |  |  |  | |
| ***IGFBP2*** | **F-5' CAATGGCGAGGAGCACTCTG** | **330** | **88.3%** | | |
|  | **R-5' TGGGGATGTGTAGGGAATAG** |  |  |  | |
| ***IGFBP3*** | **F-5' CTCAGAGCACAGACACCCA** | **335** | **95.7%** | | |
|  | **R-5' GGCATATTTGAGCTCCAC** |  |  | | |
| ***GAPDH*** | **F-5' GATTGTCAGCAATGCCTCCT** | **94** | **94.3%** | | |
|  | **R-5'GGTCATAAGTCCCTCCACGA** |  |  |  | |
| ***RN18s*** | **F-5' GAGGCCCTGTAATTGGAATGAG** | **119** | **97.2%** | | |
|  | **R-5' GCAGCAACTTTAATATACGCTATTGG** |  |  |  |  |

SUPPLEMENTAL TABLE 1. List of primer sequences used for real time PCR of sheep placentomes.
